# Putting small and big pieces together: a genome assembly approach reveals the largest Lamiid plastome in a woody vine

**DOI:** 10.7717/peerj.13207

**Published:** 2022-04-07

**Authors:** Luiz Henrique M. Fonseca, Alison G. Nazareno, Verônica A. Thode, Alexandre R. Zuntini, Lúcia G. Lohmann

**Affiliations:** 1 Instituto de Biocências, Universidade de São Paulo, São Paulo, Brazil; 2 Department of Biology, Ghent University, Ghent, Flanders, Belgium; 3 Departamento de Genética, Ecologia e Evolução, Universidade Federal de Minas Gerais, Belo Horizonte, Minas Gerais, Brazil; 4 Departamento de Botânica, Universidade Federal do Rio Grande do Sul, Porto Alegre, Rio Grande do Sul, Brazil; 5 Royal Botanic Gardens, Kew, London, United Kingdom

**Keywords:** *Bignonia magnifica*, Inverted repeats (IRs), Plastome evolution, Short- and long-reads sequencing

## Abstract

The plastid genome of flowering plants generally shows conserved structural organization, gene arrangement, and gene content. While structural reorganizations are uncommon, examples have been documented in the literature during the past years. Here we assembled the entire plastome of *Bignonia magnifica* and compared its structure and gene content with nine other Lamiid plastomes. The plastome of *B. magnifica* is composed of 183,052 bp and follows the canonical quadripartite structure, synteny, and gene composition of other angiosperms. Exceptionally large inverted repeat (IR) regions are responsible for the uncommon length of the genome. At least four events of IR expansion were observed among the seven Bignoniaceae species compared, suggesting multiple expansions of the IRs over the SC regions in the family. A comparison with 6,231 other complete plastomes of flowering plants available on GenBank revealed that the plastome of *B. magnifica* is the longest Lamiid plastome described to date. The newly generated plastid genome was used as a source of selected genes. These genes were combined with orthologous regions sampled from other species of Bignoniaceae and all gene alignments concatenated to infer a phylogeny of the family. The tree recovered is consistent with known relationships within the Bignoniaceae.

## Introduction

The plastid genome, or plastome, of flowering plants generally shows conserved structural organization, gene arrangement, and gene content (Palmer et al., 1987; Odintsova & Yurina, 2003; Green, 2011; Jansen & Ruhlman, 2012; Yurina, Sharapova & Odintsova, 2017; De Vries & Archibald, 2018). The plastome size of photosynthetic angiosperms usually ranges from 145–165 kbp (Genbank–Genome, 2021), and contains 110–130 unique genes (Jansen & Ruhlman, 2012; Yurina, Sharapova & Odintsova, 2017; De Vries & Archibald, 2018). This organelle genome typically includes a quadripartite structure that consists of a small single-copy region (SSC) with approximately 16–27 kbp, a large single-copy region (LSC) with approximately 80–90 kbp, and a pair of inverted repeats (IRs) with approximately 20–28 kbp each (Green, 2011; Jansen & Ruhlman, 2012). The typical angiosperm plastome structure has one copy of the IR flanked by the genes *ycf1* and *trnH-* GUG (IR_A_), and a second copy flanked by the genes *rps19* and *ndhF* (IR_B_; Shinozaki et al., 1986; Yukawa, Tsudzuki & Sugiura, 2005).

The angiosperm IR typically contains the entire ribosomal operon, including four ribosomal DNA (rRNA) genes encoding 4.5S, 5S, 16S, and 23S (Ruhlman & Jansen, 2014). The presence of other genes varies among angiosperms due to expansions and contractions of the IR/SC boundaries. These differences are responsible for most of the size variation observed in flowering plant plastomes (Green, 2011; Yurina, Sharapova & Odintsova, 2017). Fluctuations in IR size have been documented among closely related species (*e.g.*, Thode & Lohmann, 2019), within genera (*e.g.*, Weng, Ruhlman & Jansen, 2017), and families (*e.g.*, Guisinger et al., 2010). The contractions and expansions of the IRs are usually minor, not reaching more than a few hundred base-pairs (Goulding et al., 1996; Downie & Jansen, 2015; Wu & Chaw, 2015). Large IR expansions of more than 1,000 bp are rare and usually associated with changes in gene composition and structural rearrangements (Palmer et al., 1987; Chumley et al., 2006; Guisinger et al., 2011; Downie & Jansen, 2015; Weng, Ruhlman & Jansen, 2017).

Plastome expansions have been reported in unrelated lineages of angiosperms, such as *Annona* L. (Annonaceae, Magnoliales, with up to 201,723 bp; Blazier et al., 2016), and *Cypripedium* L. (Orchidaceae, Asparagales, with up to 212,668 bp; Guo et al., 2021). The family Geraniaceae shows a remarkable example of plastome and IR size variation, with plastid genomes ranging from 128.7 kbp in *Monsonia speciosa* L. to 242.5 kbp in *Pelargonium transvaalanse* R Knuth (Chumley et al., 2006; Guisinger et al., 2010; Weng, Ruhlman & Jansen, 2017). *Pelargonium transvaalanse* has the largest plastome known to date with IRs larger than 87.4 kbp. This plant family is also famous for extreme reconfigurations of plastid genomes, showing multiple arrangements and repeats (Guisinger et al., 2010; Weng, Ruhlman & Jansen, 2017). Despite the giant IRs, structural variation, and differences in plastome size, *Pelargonium* L’Hér and other members of Geraniaceae carry a regular number of protein-coding genes and the usual 29 tRNAs (Guisinger et al., 2011). The data available for the Geraniaceae and other flowering plant lineages suggest that these plastome increments in size and reconfigurations are not necessarily associated with relevant changes in gene expression or the overall function of the organelle.

Expansions of the IRs and multiple genomic arrangements have also been described for the tropical Tribe Bignonieae (Firetti et al., 2017; Fonseca & Lohmann, 2017; Thode & Lohmann, 2019). Bignonieae plastomes range from 155 to 158 kbp in size, although some taxa from the informally named “Multiples of Four Clade” (*i.e.,* a clade that shares multiples of four phloem wedges; see Lohmann, 2006) show significant increases in plastome size. Namely, the plastome of *Amphilophium steyermarkii* (AH Gentry) LG Lohmann is 164,786 bp long (Thode & Lohmann, 2019), while the plastome of *Anemopaegma acutifolium* DC. is 168,987 bp long (Firetti et al., 2017). Multiple independent advances of the IRa over the LSC possibly occurred within *Amphilophium* Kunth emend LG Lohmann (Thode & Lohmann, 2019; Thode, Sanmartín & Lohmann, 2019), while the IRs are identical in terms of gene composition, with differences of hundreds of bases among species in *Anemopaegma* Mart. ex Meisn. (Firetti et al., 2017). *Bignonia* is part of the “Multiples of Four Clade” however, plastomes are not available for the genus. A chloroplast genome of this clade is critical for a better understanding of the patterns and possible processes behind plastome evolution in tribe Bignonieae and the “Multiples of Four Clade.”

**Figure 1:**
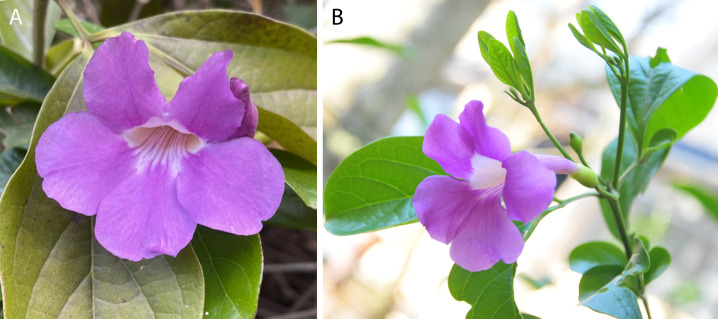
Flowers, leaves, and young branches of *Bignonia magnifica*. (A) Frontal view of the flower. (B) Young branch and leaves and the lateral view of the flower (images: A, Nemer Rahal Neto; B, Alexandre Zuntini).

**Figure 2:**
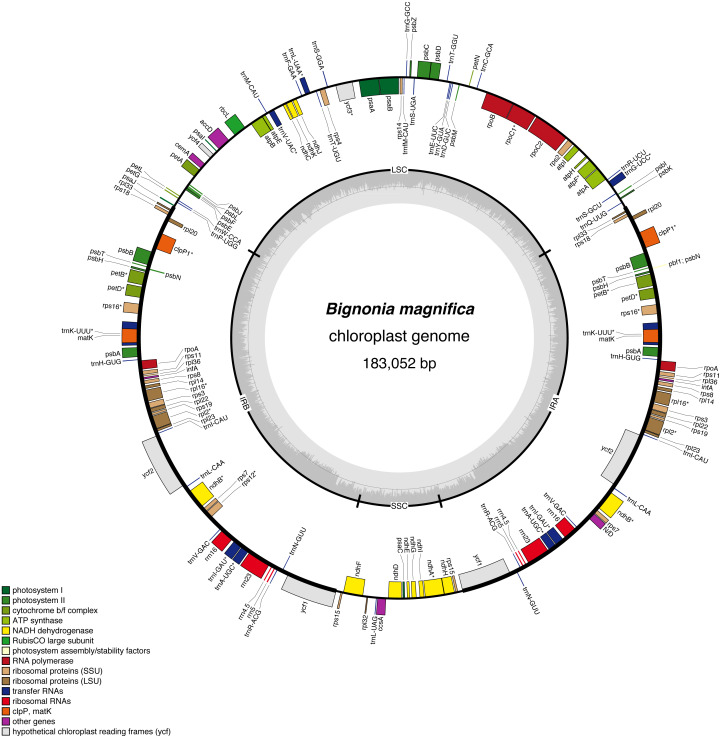
Gene map of the *Bignonia magnifica* chloroplast genome. Genes drawn inside the circle are transcribed clockwise, and those outside are transcribed counterclockwise. Genes belonging to different functional groups are color-coded. The darker gray in the inner circle corresponds to GC content, and the lighter gray corresponds to AT content. Asterisks are used to indicate genes with introns.

Here we selected *Bignonia magnifica* to sequence the first plastome of the genus (Fig. 1). This species is a tropical liana, native from Ecuador and Colombia, but widely cultivated around the world. By combining short-read Illumina data and long-read PacBio data, we assembled the whole plastome of *Bignonia magnifica* (Fig. 2). This hybrid approach has shown improved assembly accuracy to determine the plastome structure and sequence in flowering plants (Wu et al., 2014; Wang et al., 2018; Syme et al., 2020; Guo et al., 2021). Although the long reads obtained from the Pacbio platform have a higher error rate than short reads sequenced on Illumina platforms, the use of both technologies can help to reveal striking features regarding structural complexity in plastomes (Guo et al., 2021). We further compared the plastome of *B. magnifica* with genomic data available for selected species of Bignoniaceae and outgroups and evaluated the plastome size, structure, gene composition, presence of repetitive regions, and phylogenetic relationships.

## Materials and Methods

### Sampling and genome sequencing

We sampled an individual of *B. magnifica* cultivated at Instituto Plantarum (sequenced using Illumina, voucher *Lohmann 711*) and an individual cultivated at the Institute of Biosciences (IB) of the University of São Paulo (USP), São Paulo, Brazil (sequenced using PacBio, voucher *Fonseca 306*). Vouchers for each collection were deposited at the SPF herbarium (IB/USP). Approximately 60 mg of silica-dried leaflets were pulverized with Tissuelyzer® (Qiagen, Duesseldorf, Germany) for 3 min at 60 Hz. Total genomic DNA was extracted using the Invisorb® Spin Plant Mini Kit (Invitek, Berlin, Germany).

For Illumina sequencing, we followed the methodology described in Fonseca & Lohmann (2017). In short, the genomic DNA (∼5 µg) was fragmented using Covaris S-series sonicator, generating DNA fragments of around 300 bp. A genomic library was built using NEBNext DNA Library Prep Master Mix Set and the NEBNext Multiplex oligos for Illumina (New England BioLabs Inc., Ipswich, MA). The final library of *B. magnifica* was diluted to 10 nM and pooled together with other 19 non-target species in one lane and sequenced (paired-end, 2 × 100 bp) on an Illumina HiSeq2000 system (Illumina, Inc., San Diego, CA, USA) at the University of São Paulo (Escola Superior de Agricultura Luiz de Queiroz) in Piracicaba, Brazil.

For PacBio® sequencing, library preparation and sequencing followed the manufacturer’s instructions (Pacific Biosciences). In short, 10 µg of genomic DNA was isolated and fragmented to 7–20 kbp using HydroShear. A genomic library was constructed using the steps and parameters described in the manual (Pacific Biosciences). After the DNA fragment size selection, one PacBio library was constructed using SMRTbell Template Prep Kits 1.0, and one SMRT cell was sequenced by PacBio Sequel platform using Sequel™ sequencing Kit 1.2.1 at Duke Center for Genomic and Computational Biology (Duke University School of Medicine, Durham, NC, USA).

### Plastome assembly and annotation

To assemble the plastome data obtained with Illumina, we used the pipeline GetOrganelle 1.7.4.1 (Jin et al., 2020) with default parameters. Adaptors were removed and low-quality sequences trimmed using Trimmomatic 0.35 (Bolger, Lohse & Usadel, 2014) with the SLIDINGWINDOW:10:20 and MINLEN:40 parameters. Trimmed reads were used as input using the script “get_organelle_from_reads.py”, which is the main workflow of GetOrganelle (Jin et al., 2020). This script uses Bowtie2 (Langmead & Salzberg, 2012), BLAST (Camacho et al., 2009), and SPAdes 3.1.0 (Bankevich et al., 2012), as well as Python Numpy libraries and Sympy dependences. The pipeline starts mapping the reads against a database of plastomes used as seeds with Bowtie2. The initial target-associated reads were treated as “baits” to increase the number of plastome reads through multiple extension interactions. SPAdes was used to build a *de novo* FASTA assembly Graph (FASTG), and BLAST was used to remove any non-target sequences retained. The slimmed FASTG file was used to calculate all paths of the complete target organelle using the structure of the graph and coverage information.

To assemble the plastome using the data obtained through PacBio, we assembled the reads *de novo* into a number of contigs using the SMRT Analysis package 2.3 (Pacific Biosciences) with the HGAP3 parameter followed by polishing with the Quiver algorithm. A k-mer analysis was performed on each of the assemblies individually using a k-mer size of 20 using Jellyfish 2.1.3 (Marçais & Kingsford, 2011). Plastome sequences were fished out using BLAT (Kent, 2002) and the plastome of *A. arvense* as reference.

The assemblies obtained using sequences from Illumina and PacBio for *B. magnifica* were annotated using GeSeq (Tillich et al., 2017) with default parameters (https://chlorobox.mpimp-golm.mpg.de/index.html). The assemblies of *B. magnifica* were evaluated and manually compared using Geneious 9.0.2. By combining the information derived from both assemblies, we were able to establish the LSC, SSC, and IRs limits with high confidence and provide a complete plastome for *B. magnifica*. Both assemblies were largely congruent, with only small base pair differences and small indel differences (10–20 bp) observed between assemblies. Base mismatches followed the results obtained using the Illumina data, while indels followed the results obtained with PacBio. The final plastome assembly was verified using a coverage analysis implemented in Jellyfish 2.3.1 (Marçais & Kingsford, 2011). The estimate of 25-mer abundance was used to map a 25-bp sliding window of coverage across the plastome. Junctions of the quadripartite structure were tested interactively using the program afin (https://github.com/mrmckain/Fast-Plast/tree/master/afin). The final plastome map was produced using OGDRAW (Lohse, Drechsel & Bock, 2007). The NCBI accession number of the complete plastome is available in Table 1. The sequence read archive is available under BioProject number https://www.ncbi.nlm.nih.gov/bioproject/PRJNA780388.

**Table 1:** General features of the *Bignonia magnifica* and other nine Lamiid plastomes, showing number of base pairs (bp) in different genome regions (*i.e.*, LSC, large single copy; SSC, small single copy; IR, inverted repeats). The percentage of guanina-citosine (GC) and the number of genes across the IR, including protein-coding genes (CDS), ribosomal RNA (rRNA), and transfer RNA (tRNA) genes, are also presented.

Plant species	Genbank accession	Genome size (bp)	LSC (bp [%])	SSC (bp [%])	IR (bp [%])	GC (%)	IR genes (CDS/tRNA/rRNA)
*Adenocalymma pedunculatum*	MG008313	158,103	85,043 [53.8]	12,780 [8.1]	30,140 [19.1]	37.1	8/7/4
*Amphilophium paniculatum*	MK415797	163,710	76,228 [46.6]	12,738 [7.8]	37,372 [22.8]	37.7	19/7/4
*Anemopaegma arvense*	MF460829	168,806	75,194 [44.5]	12,804 [7.6]	40,404 [23.9]	37.7	20/7/4
*Bignonia magnifica*	OL470653	183,052	60,832 [33.2]	12,766 [7.0]	54,727 [29.9]	37.4	31/9/4
*Catalpa bungei*	MT591528	158,210	84,928 [53.7]	12,664 [8.0]	30,309 [19.2]	38.1	8/7/4
*Dolichandra cynanchoides*	MG831874	158,110	85,595 [54.1]	12,753 [8.1]	30,382 [19.2]	38.1	8/7/4
*Lippia origanoides*	MK248831	154,310	85,119 [55.2]	17,291 [11.2]	25,979 [16.8]	39.2	8/7/4
*Pyrostegia venusta*	MG831878	165,158	74,040 [44.8]	12,713 [7.7]	39,137 [23.7]	38.0	20/7/4
*Tabebuia nodosa*	MT447061	158,454	85,437 [53.9]	12,785 [8.1]	30,116 [19.0]	38.2	8/7/4
*Tecomaria capensis*	MG831880	153,263	85,049 [55.5]	18,898 [12.3]	24,658 [16.1]	38.1	7/7/4

### Tandem repeat detection

Phobos 3.3.12 (http://www.rub.de/ecoevo/cm/cm_phobos.htm) was used *via* Geneious to search, count, and annotate tandem repeats. The “exact search” option was used, searching for tandem repeats between 1 and 1,000 bp long. Default values were used for all other parameters. The “remove hidden repeats” setting was enabled. The results obtained for *B. magnifica* were compared with those obtained for eight selected species of Bignoniaceae in previous studies: (i) *Adenocalymma pedunculatum* (Vell.) LG Lohmann (Fonseca & Lohmann, 2017), (ii) *Amphilophium paniculatum* (L.) Kunth (Thode & Lohmann, 2019), (iii) *Anemopaegma arvense* (Vell.) Stellfeld ex JF Souza (Firetti et al., 2017), (iv) *Catalpa bungei* CA Mey (Li et al., 2020), (v) *Dolichandra cynanchoides* Cham. (Fonseca & Lohmann, 2017), (vi) *Pyrostegia venusta* (Ker Gawl.) Miers (Fonseca & Lohmann, 2018), (vii) *Tabebuia nodosa* (Griseb.) Griseb. (Fonseca & Lohmann, 2020), and (viii) *Tecomaria capensis* (Thunb.) Spach (Fonseca & Lohmann, 2018). The plastome of *Lippia origanoides* Kunth (Verbenaceae) was also used for comparison (NCBI accession numbers at Table 1; Sarzi et al., 2019). Verbenaceae is now being consistently recovered as the sister family of Bignoniaceae (Fonseca, 2021), representing a good outgroup for comparative studies.

### Comparative analyses of plastomes

To determine synteny, we compared the plastome of *B. magnifica* with plastomes of the eight selected Bignoniaceae species and that of *L. origanoides*. This analysis was performed in MAUVE 2.4.0 (Darling, Mau & Perna, 2010) using progressiveMauve as the alignment algorithm, and MUSCLE 3.6 (Edgar, 2004) as the internal aligner with default parameters. For each sample, only the IRa was maintained. The plastome size of *B. magnifica* was also compared with the 6,231 complete angiosperm plastomes available on GenBank (Genbank–genome, Sep. 2021). Plastome size information was compiled by angiosperm order.

### Phylogenetic analyses

To infer the phylogenetic placement of *B. magnifica* within the Bignoniaceae, we used the same 80 plastid genes (Table 1) used to infer the phylogeny of angiosperms by Li et al. (2019). DNA sequences were aligned using MAFFT 7.309 (Katoh & Standley, 2013). The alignments were edited using GBlocks (Talavera & Castresana, 2007) and regions found in less than 50% of the species were deleted. PartitionFinder2 (Stamatakis, 2006; Lanfear et al., 2014; Lanfear et al., 2016) was used to estimate partition schemes and molecular evolutionary models for each of the 80 plastid genes. Phylogenetic reconstructions were conducted using 12 species. Regions obtained from the newly assembled plastome were combined with sequences from the nine other species used here for structural comparisons, plus two other species with plastid genomes available and used as outgroups, *Sesamum indicum* L. (Pedaliaceae) and *Scrophularia dentata* Royle ex Benth. (Scrophulariaceae). Phylogenetic inferences were conducted using Maximum Likelihood (ML) in RAxML 8.2.9 (Stamatakis, 2014), and Bayesian Inference (BI) with MrBayes 3.2 (Ronquist et al., 2012). Branch support for ML was estimated using 1,000 bootstrap replicates (bs) and for BI using posterior probabilities (pp).

## Results

### Plastome assembly

We sequenced the complete plastome of *B. magnifica* using Illumina and PacBio technologies. For the sequences generated with Illumina, we obtained 3,563,896 paired-end reads after the adaptors were removed and low-quality sequences trimmed. For R1 and R2, we obtained 352,991 and 59,697 non-paired reads, respectively. The mean length read was 81.8 bp long. After running the pipeline GetOrganelle, the maximum contig obtained was 182,643 bp, corresponding to almost the entire plastome sequence. The PacBio sequencing generated 150,292 (1.02 Gb) single-molecule long subreads in total, with an average length of 6,777 bp, and N50 of 14,789 bp. Overall, 172 contigs were identified, the longest of which was 151,204 bp in length, and represented the partial plastome with one copy of the IR. Results obtained using both sequencing technologies were visually compared to reach the final plastome size and evaluate the boundaries between LSC, SSC, and IRs. Junctions of the quadripartite structure were tested interactively and recovered in all analyses. The mean plastome coverage obtained using Illumina data was 825.8×, with more than 99% of the bases with coverage equal or larger than 100×.

### Plastome features

The final plastome size of *B. magnifica* is 183,052 bp long. The genome has the typical quadripartite structure of angiosperms, which consists of a pair of IR regions (54,727 bp), a LSC region (60,832 bp), and an SSC region (12,766 bp) (Table 1). A circular plastome map of *B. magnifica* is shown in Fig. 2. The average GC content is 37.4%. The plastome includes 157 genes, representing 110 coding regions, 39 tRNAs, and eight rRNA (Table 1, Table 2). Thirteen different genes have at least one intron, while three genes have two introns (*i.e., clpP, rps12*, and *ycf3*) (Table 2). For *rps12*, a trans-splicing event was observed with the 5′ end located in LSC, and a duplicated *rps12* 3′ in the IRs (Table 2). Among protein-coding genes, 122 started with the standard initiator AUG. The *rps19* and *ndhD* are exceptions, with *rps19* starting with GUG and *ndhD* starting with ACG. The stop codon UAA was the most common, followed by UAG and UGA.

**Table 2:** Plastome gene content and functional classification in *Bignonia magnifica*.

Gene function	Gene type	Gene
Self-replication	rRNA genes	*rrn4.5*^a^, *rrn5*^a^, *rrn16*^a^, *rrn23*^a^
	tRNA genes	*trnA-UGC*^a,*^, trnC-GCA, trnD-GUC, trnE-UUC, trnF-GAA, trnfM-CAU, trnG-UCC, trnG-UCC^*^, *trnH-GUG*^a^, *trnI-CAU*^a^, *trnI-GAU*^a^, *trnK-UUU*^a^, *trnL-CAA*^a^, *trnL-UAA*, *trnL-UAG, trnM-CAU, trnN-GUU*^a^, *trnP-UGG, trnQ-UUG, trnR-ACG*^a^*trnR-UCU, trnS-GCU, trnS-GGA, trnS-UGA, trnT-GGU, trnT-UGU, trnV-GAC*^a^, *trnV-UAC, trnW-CCA, trnY-GUA*
	Small ribosomal subunit	
	Large ribosomal subunit	
	DNA dependent RNA	*rps2, rps3*^a^, *rps4, rps7*^a^, *rps8*^a^, *rps11*^a^, *rps12*^a,**^, *rps14, rps15*^a^, rps16^*,a^, *rps18*^a^, *rps19*^a^
		*rpl2*^a,*^, *rpl14*^a^, *rpl16*^a,*^, *rpl20*^a^, *rpl22*^a^, *rpl23*^a^, *rpl32, rpl33*^a^, *rpl36*^a^, *rpoA*^a^, rpoB, rpoC1^*^, *rpoC2*
Photosynthesis	Photosystem I	*psaA*, psaB, psaC, psaI, psaJ, ycf3^**^
	Photosystem I	*psbA*^a^, *psbB*^a^, *psbC, psbD, psbE, psbF, psbH*^a^, *psbI, psbJ, psbK, psbL, psbM, psbN*^a^, *psbT*^a^, *psbZ*
	NADH-dehydrogenase	ndhA^*^, *ndhB*^a,*^, *ndhC, ndhD, ndhE, ndhF, ndhG, ndhH, ndhI, ndhJ, ndhK*
	Cytochrome b6/f complex	*petA, petB*^a,*^, *petD*^a,*^, *petG, petL, petN*
	ATP synthase	atpA, atpB, atpE, atpF^*^, *atpH, atpI*
	Rubisco	*rbcL*
Other genes	Translational initiator	*infA* ^a^
	Maturase	*matK* ^a^
	Protease	*clpP* ^a,**^
	Envelope membrane protein	*cemA*
	Subunit of acetil-CoA-carboxylase	*accD*
	c-type cytochrome synthesis	*ccsA*
Unknown function	Conserved open read frames	*ycf1*^a^, *ycf2*^a^, *ycf4*^b^

**Notes.**

* Gene with one intron.

** Gene with two introns.

a Gene with two copies.

We identified 688 repetitive motifs for *B. magnifica* using Phobos. These tandem regions ranged from a single nucleotide repetition (mononucleotide) to 52-nucleotide repetitive regions. These regions represented 6,6% of the total genome size. Among all species analyzed, *L. origanoides* showed fewer repetitive regions (567 in total), while *A. arvense* showed the highest number of repetitive regions (694 in total). The number of each class of repetitive region (*i.e.,* mononucleotide, dinucleotide, or trinucleotide) differed among species but showed similar numbers within the Bignoniaceae. Mononucleotide repetitive regions were the most common among Bignoniaceae species, while dinucleotide repetitions were the least common. The same pattern was observed in *L. origanoides*, although a lower number of repetitive regions was observed for each class (Table 3).

**Table 3:** Total number (*T*) and genome length percentage (%) of perfect tandem repeats composed of motifs of 1–100 bp in *Bignonia magnifica* and other nine Lamiid plastomes.

Plant species	*T*	%	Number of simple sequence repeats						
			Mono	Di	Tri	Tetra	Penta	10 ≥ × > 5	100 ≥ × > 10
*Adenocalymma pedunculatum*	689	7.1	252	24	65	75	71	181	19
*Amphilophium paniculatum*	686	6.2	253	27	61	69	81	172	22
*Anemopaegma arvense*	694	7.1	269	26	55	72	72	166	30
*Bignonia magnifica*	688	6.6	264	22	59	74	77	166	26
*Catalpa bungei*	664	5.7	242	27	61	84	66	171	13
*Dolichandra cynanchoides*	676	6.2	249	28	59	81	69	168	22
*Lippia origanoides*	567	4.9	212	19	54	55	62	150	15
*Pyrostegia venusta*	649	6.2	256	21	55	70	68	153	26
*Tabebuia nodosa*	655	6.0	237	26	57	73	74	160	28
*Tecomaria capensis*	661	5.9	263	24	58	75	65	157	19

### Comparative plastome structure and size

**Figure 3:**
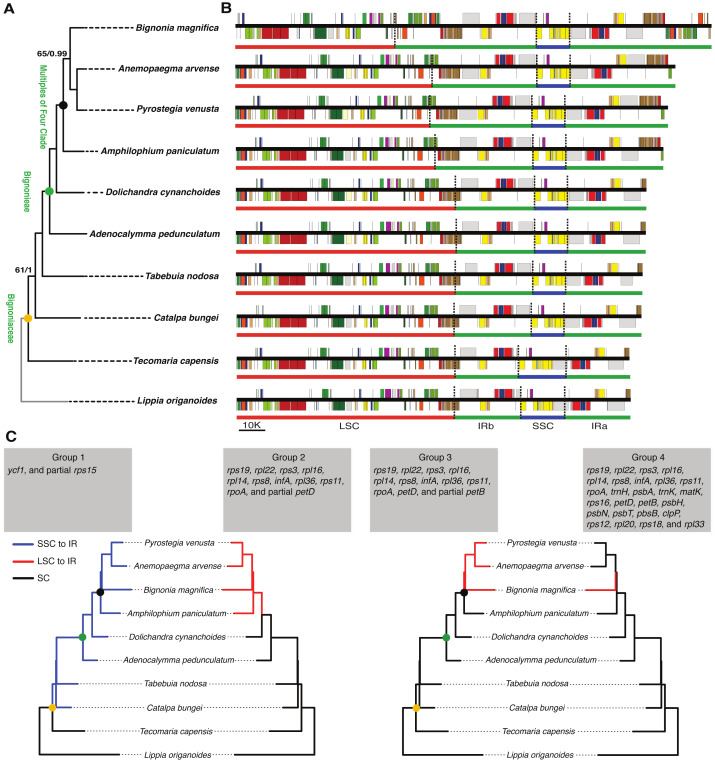
Phylogenetic relationships, and comparison of plastome structure among nine species of Bignoniaceae and *Lippia origanoides*. (A) Phylogenetic tree obtained using 80 plastome protein coding regions. Maximum values of support were omitted. (B) Linear plastid maps. (C) Phylogenetic distribution of inverted repeat (IR) inclusion and exclusion. The Branches where the gene or gene group were located in SSC or LSC are highlighted in blue and red, respectively.

**Figure 4:**
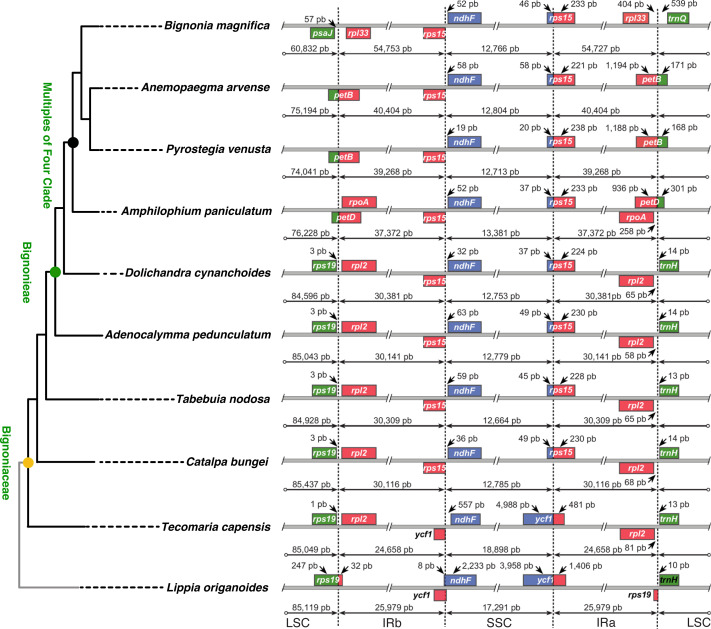
Comparisons of the large single copy (LSC), small single copy (SSC), and inverted repeated (IR) region borders among nine species of Bignoniaceae and *Lippia origanoides* chloroplast genomes. Genes shown above the lines are transcribed forward while genes shown below the lines are transcribed reversely. Two-headed arrows indicate plastome partition sizes in base pairs and single-headed arrows indicate size of features or distances between plastome partition borders and features.

Differences in the IR/SSC boundary region were observed when the plastome of *B. magnifica* was compared to the plastomes of *L. origanoides* and *T. capensis*. These differences suggest a reduction of the SSC due to the incorporation of the entire *ycf1* gene and part of the *rps15* gene into the IRs in the clade composed of *C. bungei*, *T. nodosa*, *A. pedunculatum*, *D. cynanchoides*, *A. paniculatum*, *B. magnifica*, *A. arvense*, *P. venusta* (Group 1; Figs. 3 and 4). Successive expansions of the IR over the LSC were also observed within the “Multiples of Four Clade” of Tribe Bignonieae (Lohmann, 2006) (Figs. 3 and 4). At least three gene translocations occurred inside the clade as follows: (i) the genes *rps19, rpl22, rps3, rpl16, rpl14, rps8, infA, rpl36, rps11, rpoA,* and the partial *petD* were incorporated in the IRs of the clade (*A. paniculatum, B. magnifica*, *A. arvense*, *P. venusta*) (Group 2); (ii) the entire gene *petD* and the partial *petB* gene were incorporated in the IRs of the clade (*B. magnifica*, *A. arvense*, *P. venusta*) (Group 3); and (iii) the entire gene *petB* and the genes *psbH, psbN, psbT, psbB, clpP, rps12, rpl20, rps18*, and *rpl33* were incorporated in the IRs of *B. magnifica* (Group 4; Figs. 3 and 4).

The incorporation of genes within the IRs led to a massive increase in size of the plastomes of *B. magnifica* and other members of the “Multiples of Four Clade.” As described above, at least four gene translocations due to IR expansion occurred (Figs. 3 and 4). The first movement was observed in the SSC-IRa boundary. The second and third movements changed the structure of the LSC-IRb boundary. Group 2 also incorporated part of the gene *petD*, while Group 3 incorporated the entire *petD* and part of the *petB* genes as part of the IR. The fourth movement combined expansions at both the LSC-IRa and LSC-IRb boundaries (Figs. 3 and 4). The boundary between SSC-IRb is constant within Bignoniaceae. Structural differences were observed between members of the Bignoniaceae and the plastome of *L. origanoides* (Fig. 4). While this difference suggests a fifth transition in SS-IR boundaries, the shift of the SSC-IRb boundary towards IRb and the incorporation of the *ndhF* within the SSC region was not observed in two other Verbenaceae plastomes available (*Aloysia citriodora* Ortega ex Pers (NC034695) and *Verbena officinalis* L. (MW328640)), suggesting that this transition is exclusive of *L. origanoides* or exclusive to an internal clade of the family.

No differences in gene order were observed within *B. magnifica*, when compared to the other Bignonieae plastomes analyzed. While an apparent change in synteny was observed for *B. magnifica*, this gene block movement seems to have been caused by the incorporation of genes close to the LSC-IRa boundary by the IR (Fig. S1). Rearrangements were observed for the Bignonieae clade composed of *A. arvense* and *P. venusta*. The inversion of the region containing the genes *ycf2, trnI*, and *trnL* is consistent with earlier findings for *Anemopaegma* as a whole (Firetti et al., 2017).

**Figure 5:**
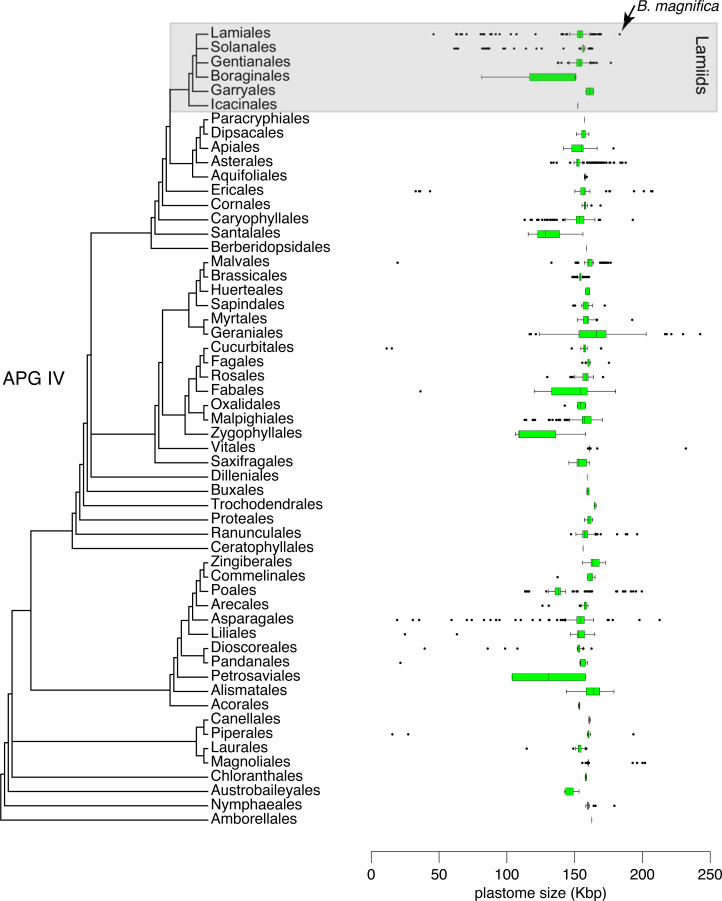
Distribution of 6,231 plastid genome sizes through the flowering plant phylogeny.

A size comparison of all 6,231 angiosperm complete plastomes available in GenBank, including 724 plastomes of Lamiids (Genbank–Genome, Sep. 2021), indicated that the plastome of *B. magnifica* is the largest Lamiid plastome sequenced to date (Fig. 5). Within the Lamiids, the plastome of *B. magnifica* was followed in size by the genome of *Hoya carnosa* (Apocynaceae), with 176,340 bp, and eight other *Anemopaegma* plastomes. Forty other angiosperm plastomes were larger than the plastome of *B. magnifica*. These plastomes are distributed through various angiosperm clades, belonging to the following orders: Asparagales (*e.g.*, *Cypropedium* L.), Asterales (*e.g.*, *Cyphia* P.J. Bergius), Caryophyllales (*Drosera rotundifolia* L.), Ericales (*e.g.*, *Rhododendron* L.), Geraniales (*e.g.*, *Pelargonium*), Magnoliales (*e.g.*, *Annona*), Myrtales (*Eucalyptus* L’Hér), Piperales (*Asarum* L.), Poales (*e.g.*, *Eleocharis* R. Br.) , Ranunculales (*Corydalis* DC.), and Vitales (*Vitis romanetti* Rom. Caill.) (Fig. 4).

### Phylogenetic analyses

The phylogenetic hypotheses reconstructed with Maximum Likelihood and Bayesian Inference using a dataset composed of 80 plastome genes and 12 species showed identical topologies. The outgroups *S. indicum* L. and *S. dentata* were used to root the trees. The family Bignoniaceae, the Tribe Bignonieae, and the “Multiples of Four Clade” emerged as monophyletic, confirming earlier phylogenetic findings (Lohmann, 2006; Olmstead et al., 2009). The position of *B. magnifica* as part of the “Multiples of Four Clade” was also recovered (Lohmann, 2006). While all clades showed maximum or high support in the Bayesian analysis, two relationships were poorly supported in the ML analysis: (i) the sister-group relationship between *Catalpa* and a clade composed of Bignonieae + *Tabebuia nodosa* (61 bs); and (ii) the Bignonieae clade composed of *B. magnifica, A. arvense*, and *P. venusta* (65 bs; Fig. 2). These relationships were also poorly supported in earlier studies (Lohmann, 2006; Olmstead et al., 2009), suggesting recalcitrant points in the phylogeny.

## Discussion

In this study, we assembled the plastome of *Bignonia magnifica* and compared it with nine other Lamiid plastomes. The recovered *B. magnifica* plastome follows the canonical quadripartite structure, synteny, and gene composition found in other angiosperm plastomes. The total number of repetitive regions and the number observed for each class of repetitive regions is similar to that observed in other species of the family. Remarkable differences were observed in the size of the IRs, the longest in the family, and responsible for the largest plastome available to date for the entire Lamiids. Five events of IR expansion were observed within the eight Bignoniaceae species compared, suggesting multiple expansions of the IRs over the SC regions. The newly generated plastid genome was used as a source of selected genes. These genes were combined with orthologous regions sampled from other species of Bignoniaceae and all gene alignments concatenated to infer a phylogeny of the family. The tree reconstructed here recovered a monophyletic Bignoniaceae and a monophyletic tribe Bignonieae, corroborating previous findings. The topology recovered here also confirmed the monophyly of the “Multiples of Four Clade,” and previously recovered relationships within this lineage.

### Recapitulating earlier findings

Plastome architecture, arrangement, and gene content are highly conserved among seed plants (Palmer et al., 1987; Odintsova & Yurina, 2003; Green, 2011; Jansen & Ruhlman, 2012). The plastome of *B. magnifica* is another example of canonical architecture, with a quadripartite structure and the variation in gene position related to IR expansions. No rearrangements were observed when the plastome of *B. magnifica* was compared to other plastomes from the family Bignoniaceae, Tribe Bignonieae, or the “Multiples of Four Clade” (Fig. S1). Gene-content also follows other photosynthetic flowering plants (Table 2; Jansen & Ruhlman, 2012).

The total number of repetitive regions or the number of repetitive regions within each class was also similar among the species analyzed (Table 3). Repetitive sequences and IR expansions are correlated and involved in syntenic disruptions of plastomes (Chumley et al., 2006). Here the IR expansion of *B. magnifica* could not be associated with massive structural disruptions (Fig. S1), nor with an increase in the number of repetitive regions (Table 3). This result was also observed in other members of the “Multiple of Four Clade” sampled (Firetti et al., 2017; Thode & Lohmann, 2019), suggesting that the IR expansions within the clade are not leading to increases in the number of repetitive regions nor to the accumulation of rearrangements.

The phylogeny recovered here is congruent with a previous phylogeny of the Bignoniaceae (Olmstead et al., 2009), and a previous phylogeny of Bignonieae that placed *B. magnifica* within the “Multiples of Four Clade” (Lohmann, 2006). The phylogeny reconstructed here aimed to provide an evolutionary framework within which to compare selected Bignoniaceae plastomes. While sampling is reduced, inflating the support of most nodes, and simplifying the possible implications of the results, two nodes were poorly supported, including the node leading to the clade composed of *A. arvense, P. venusta*, and *B. magnifica* (Fig. 2). Recalcitrant branches were previously observed in *Adenocalymma* (Fonseca & Lohmann, 2018) and *Amphilophium* (Thode, Sanmartín & Lohmann, 2019) illustrating some limitations of plastome data for phylogeny reconstruction.

### The making of large plastomes

The plastome of *B. magnifica* recovered in this study is the largest known to date for the entire Lamiid (Fig. 4). A dramatic increase in IR size led to a plastid genome with 183,052 bp, which is 14,065 bp longer than the plastome of *Anemopaegma acutifolium* DC., the second largest in the Bignoniaceae. Plastome size increase due to IR expansion over the LSC regions has been described for *Anemopaegma* (Firetti et al., 2017) and *Amphilophium* (Thode & Lohmann, 2019). These two genera, as well as *Bignonia, Mansoa*, and *Pyrostegia*, are part of the “Multiples of Four Clade” (Lohmann, 2006). Within the clade, at least three events of plastome increase were observed (Fig. 3). The expansions of the IR over the LSC region observed for *B. magnifica* involved the capture of LSC regions from both LSC/IRa and LSC/IRb boundaries and likely resulted from independent IR expansions (Fig. 3).

The results obtained here bring new insights into plastome evolution. However, the elucidation of the exact number, mechanism, and when those expansions occurred throughout the clade requires an improved sampling of plastomes within Tribe Bignonieae and the “Multiples of Four Clade”. In total, ten *Anemopaegma* plastomes are available, all of which are homogenous in terms of structure and size; however, a higher number of *Anemopaegma* plastomes is needed so generalizations can be made (Firetti et al., 2017). Differences in plastome size and IR expansions were observed among the 11 complete *Amphilophium* plastomes sequenced to date (Thode & Lohmann, 2019); however, the species sharing structural patterns are not necessarily closely related and no clear phylogenetic pattern was observed (Thode, Sanmartín & Lohmann, 2019).

While the plastome of *B. magnifica* is giant within Bignonieae and other Lamiids, 40 other plastomes from diverse angiosperm clades are larger than the plastome of *B. magnifica* (Fig. 5; Guisinger et al., 2010; Weng, Ruhlman & Jansen, 2017). Almost all of these plastomes share the expansions of the IRs over SC regions as the main mechanism responsible for their large sizes (Blazier et al., 2016; Zhang et al., 2016; Weng, Ruhlman & Jansen, 2017; Ruhlman & Jansen, 2018; Lee, Ruhlman & Jansen, 2020). These findings highlight the importance of these expansions for plastid genome size and gene composition. As these expansions of the IRs are found throughout the “Multiples of Four Clade”, more plastomes with expansions are expected, some of which might be larger than the plastome of *B. magnifica* documented here.

Expansions of IRs are linked to some plastome properties, such as the number of repetitive regions and the frequency of rearrangements (Guisinger et al., 2011; Weng, Ruhlman & Jansen, 2017; Lee, Ruhlman & Jansen, 2020). No significant differences were observed when we compared the plastome of *B. magnifica* with nine other genomes in terms of the number of repetitive regions and its synteny (Table 3, Fig. S1). Improved sampling within the “Multiples of Four Clade” would allow statistical testing and the implementation of comparative methods to evaluate putative correlations between plastome size, DNA sequence, and structural properties (Weng, Ruhlman & Jansen, 2017).

The reduction of substitution rates on genes in the IR (when compared to SC genes) is also worth noting (Zhu et al., 2016; Weng, Ruhlman & Jansen, 2017). The two identical IR copies provide a template for error correction when a mutation occurs in one of the copies, likely suppressing substitution rates in the IR. When the IRs incorporate genes, substitution rates are expected to decrease in those regions. While this expectation was tested in *Pelargonium*, no significant correlations were found (Weng, Ruhlman & Jansen, 2017). These findings illustrate that the effect of IR expansion/contraction on substitution rates may not be relevant or easily detectable. New molecular data on *Pelargonium* and other plant groups are necessary to properly test this prediction. The diversity of plastomes found within the “Multiples of Four Clade” makes this group an excellent model within which to test hypotheses about plastome evolution.

## Conclusion

The complete plastome of *B. magnifica* showed the striking dimensions that these genomes can reach within the family, especially within Tribe Bignonieae. Some patterns were recovered when plastomes are compared in lineages with IR expansions, however, rigorous tests are still necessary to formally evaluate the patterns encountered and the putative underlying causes. Indeed, new data is still needed to answer many open questions, such as: (i) Are these expansions or contractions related to plastome rearrangements? (ii) Are the expansions or contractions related to an increase or decrease in the number of repetitive regions? (iii) Is it possible to observe differences in substitution rates for genes found in different compartments of the genome? The dozens of complete plastomes available for the Tribe Bignonieae to date (Firetti et al., 2017; Fonseca & Lohmann, 2017; Thode & Lohmann, 2019) contribute important data and bring new insights into the molecular patterns. The extensive phylogenetic data available (Firetti et al., 2017; Fonseca & Lohmann, 2018; Thode, Sanmartín & Lohmann, 2019) or to be published soon, combined with more complete plastomes for members of Bignonieae provide a strong basis for future studies on plastome evolution in the clade. In this sense, the plastome of *Bignonia magnifica* is a significant step forward, showing new molecular patterns inside tribe Bignonieae.

## Supplemental Information

10.7717/peerj.13207/supp-1 Supplemental Information 1 Plastome sequence of *Bignonia magnifica*

10.7717/peerj.13207/supp-2 Supplemental Information 2 Supplemental material

## Additional Information and Declarations

### DNA Deposition

The following information was supplied regarding the deposition of DNA sequences: The plastome is available at GenBank: OL470653.

### Data Availability

The following information was supplied regarding data availability: Data is available at NCBI SRA data: PRJNA780388.
