# Supplementary material for: Putting small and big pieces together: a genome assembly approach reveals the largest Lamiid plastome in a woody vine
Source: PeerJ. 2022 Apr 7;10:e13207. doi: 10.7717/peerj.13207 (PMC8995027; doi:10.7717/peerj.13207)
Supplement: Supplemental Information 2 [file peerj-10-13207-s002.docx]

**Figure S1.** MAUVE alignment of plastid genomes of Bignonieae. Within each of the linear genomes, local collinear blocks are represented by blocks of the same color connected by lines.
